# Syntaphilin-Mediated Docking of Mitochondria at the Growth Cone Is Dispensable for Axon Elongation *In Vivo*

**DOI:** 10.1523/ENEURO.0026-19.2019

**Published:** 2019-09-17

**Authors:** Tine Verreet, Cory J. Weaver, Hiromu Hino, Masahiko Hibi, Fabienne E. Poulain

**Affiliations:** 1 Department of Biological Sciences, University of South Carolina, Columbia, South Carolina 29208; 2 Laboratory of Organogenesis and Organ Function, Bioscience and Biotechnology Center, Nagoya University, Nagoya 464-8601, Aichi, Japan

**Keywords:** axon growth, confocal live imaging, mitochondria, Syntaphilin, visual system, zebrafish

## Abstract

Mitochondria are abundantly detected at the growth cone, the dynamic distal tip of developing axons that directs growth and guidance. It is, however, poorly understood how mitochondrial dynamics relate to growth cone behavior *in vivo*, and which mechanisms are responsible for anchoring mitochondria at the growth cone during axon pathfinding. Here, we show that in retinal axons elongating along the optic tract in zebrafish, mitochondria accumulate in the central area of the growth cone and are occasionally observed in filopodia extending from the growth cone periphery. Mitochondrial behavior at the growth cone *in vivo* is dynamic, with mitochondrial positioning and anterograde transport strongly correlating with growth cone behavior and axon outgrowth. Using novel zebrafish mutant lines that lack the mitochondrial anchoring proteins Syntaphilin a and b, we further show that Syntaphilins contribute to mitochondrial immobilization at the growth cone. Syntaphilins are, however, not required for proper growth cone morphology and axon growth *in vivo*, indicating that Syntaphilin-mediated anchoring of mitochondria at the growth cone plays only a minor role in elongating axons.

## Significance Statement

Proper axon elongation and pathfinding are essential for nervous system wiring. The growth cone, a dynamic structure at the distal end of axons, mediates axonal growth and guidance. Here, we describe for the first time *in vivo* the behavior of mitochondria at the growth cone of elongating axons. We show that mitochondria accumulate in the growth cone central area and are also present in its periphery. We further provide evidence that Syntaphilin, which immobilizes mitochondria along mature axons, also docks mitochondria at the growth cone. However, the loss of Syntaphilin did not cause a complete depletion of mitochondria from the growth cone and did not affect axon elongation, indicating that other mitochondria-docking factors regulate axon growth during development.

## Introduction

Nervous system formation and function critically rely on mitochondria. The ability of mitochondria to produce ATP via oxidative phosphorylation and to buffer cytosolic calcium is especially important in neurons that have a high energy demand and require proper ion homeostasis. Mitochondrial dynamics, including transport, fission, and fusion, contribute to the correct distribution of mitochondria in axons and are therefore essential regulators of mitochondrial functions (Trevisan et al., 2018). Mitochondria are targeted to regions distant from the cell body, such as the axonal growth cone and synaptic terminals, through their active transport along microtubules (Melkov and Abdu, 2018). Overall, the importance of mitochondrial dynamics for proper neuronal development and function is emphasized by the large number of neurologic disorders caused by mutations affecting mitochondrial proteins (Misko et al., 2010; Bertholet et al., 2016).

During development, distal growing axons and growth cones contain higher densities of mitochondria compared with proximal axonal regions (Morris and Hollenbeck, 1993). This asymmetrical distribution is thought to be necessary for axon growth (Smith and Gallo, 2018). For instance, *in vitro*, increasing mitochondrial density in the distal axon and growth cone by overexpressing the mitochondrial biogenesis regulator peroxisome proliferator-activated receptor gamma coactivator-1α (PGC-1α) was shown to increase axonal length (Vaarmann et al., 2016). Other studies have reported that factors promoting or inhibiting axon growth regulate mitochondrial localization at the growth cone (Beck et al., 2012; Sainath et al., 2017). For example, adding the growth-promoting factor BDNF to cultured neurons increases distal mitochondrial density, whereas adding the repulsive guidance cue ephrin-A5 or using chondroitin sulfate proteoglycans as a nonpermissive substrate both cause mitochondria to leave the growth cone (Beck et al., 2012; Sainath et al., 2017). While these *in vitro* studies have highlighted a significant role for mitochondria in axon extension and growth cone morphology, mitochondrial dynamics at the growth cone during axon pathfinding have never been assessed *in vivo*.

The mechanisms responsible for maintaining mitochondria in distal growing axons are not yet fully understood. Mitochondria are able to attach to both microtubule and actin cytoskeletons (Boldogh and Pon, 2006), and could therefore be immobilized on microtubules in the growth cone central area after being transported along developing axons. Such stalling might be mediated by Syntaphilin (Snph), a mitochondrial membrane protein able to directly tether mitochondria to microtubules, thereby stalling mitochondrial transport (Kang et al., 2008). By immobilizing mitochondria, Snph was shown to reduce synaptic plasticity (Kang et al., 2008) and modulate axon branching (Courchet et al., 2013). Snph was further found to prevent mitochondrial transport toward the distal axon and inhibit axon regeneration after injury (Zhou et al., 2016). However, whether Snph also docks mitochondria at the growth cone during axon elongation has never been tested.

The zebrafish embryo offers a unique accessibility and transparency to monitor mitochondrial distribution and transport along axons *in vivo* (Plucińska et al., 2012; Campbell et al., 2014; Drerup et al., 2017; Wehnekamp et al., 2019). Here, we used time-lapse imaging of mitochondria in single retinal axons as they elongate along the optic tract in zebrafish to characterize for the first the time *in vivo* mitochondrial dynamics at the growth cone during axon pathfinding. We show that mitochondrial distribution at the growth cone correlates with axon growth status, and that mitochondrial transport toward the growth cone correlates significantly with axon elongation. We further demonstrate that Snph contributes to mitochondrial docking at the growth cone during axon pathfinding. However, growth cone morphology and axon elongation are unaffected in *snph* mutants, indicating that the direct anchoring of mitochondria to growth cone microtubules by Snph only plays a minor role in axon elongation.

## Materials and Methods

### Zebrafish husbandry

All animal procedures were performed in accordance with the University of South Carolina’s Institutional Animal Care and Use Committee (IACUC). Zebrafish (*Danio rerio*) wild-type (WT; AB strain) and *lakritz* (*lak*) mutant (Kay et al., 2001) embryos were obtained from natural matings, raised at 28.5˚C in E3 medium (5 mm NaCl, 0.17 mm KCl, 0.33 mm CaCl_2_, and 0.33 mm MgSO_4_) in the presence of 150 mm 1-phenyl-2-thiourea (PTU; Sigma-Aldrich) to prevent pigment formation, and staged by age and morphology (Kimmel et al., 1995). Zebrafish possess a polygenic sex determination system, and sex-associated chromosomal regions are not fixed for the species (Liew et al., 2012). Our experiments were conducted on embryos before the onset of sexual differentiation, which occurs only at ∼2.5 months after metamorphosis is complete (Maack and Segner, 2003).

### TALEN-mediated mutagenesis and genotyping of *Snph* mutants

The transcription activator-like effector nucleases (TALENs) to target zebrafish *snpha* and *snphb* were designed using TAL Effector Nucleotide Targeter 2.0 (https://tale-nt.cac.cornell.edu/node/add/talen; Cermak et al., 2011). The TALENs for *snpha* contained the following repeat variable di-residues (RVDs): HD NI HD NN HD HD NI HD NG NN HD NI NN HD NI NN and NI NN HD HD NN NG NN HD NI HD NN NG NI NN NI NG NN HD, which targeted the sequences CACGCCACTGCAGCAG and AGCCGTGCACGTAGATGC, respectively. The TALENs for *snphb* contained the following RVDs: NN NI NN HD NI NI NG NI HD HD NG HD NI HD HD HD and NN HD HD NG NN NI NG NN HD NI HD NI HD HD NG HD, which targeted GAGCAATACCTCACCC and GCCTGATGCACACCTC, respectively. The TALEN cDNAs were assembled as described previously (Sakuma et al., 2013) and subcloned into pCS2pTAL3DD and pCS2pTALRR (Dahlem et al., 2012). Capped RNAs were synthesized from NotI-digested TALEN expression plasmids using SP6 RNA polymerase (Promega) in the presence of m´G(5´)ppp(5´)G RNA Cap Structure Analog (NEB). One nanoliter of solution containing a pair of TALEN RNAs (0.4 μg/μl each) was injected into zebrafish embryos at the one-cell stage. Deletion mutations in the target region were detected using heteroduplex mobility analysis (Ota et al., 2013). The following primers were used: AGAATCATGGCATTCGTCCTC and TGAAGCCTCTCCACATTTTCTT to detect the 14 bp deletion in *snpha*; and AATGATAACCATGGCATTCGAC and CTTTAAGCCGTGCTCTCAGGT to detect the 4 bp deletion in *snphb*. PCR products were separated on 12% or 20% Tris-borate-EDTA acrylamide gels or on a 4% Metaphor gel (Lonza). The *snphb* mutation was additionally detected using high-resolution melting analysis (Parant et al., 2009) using the following primers: AGCAATACCTCACTCCACTG and GCCTGATGCACACCTCTTTC.

### Cloning of *snpha* and *snphb* coding sequences

mRNA from embryos at 24 h postfertilization (hpf) was isolated using TRIzol (Thermo Fisher Scientific) and the RNeasy Mini Kit (Qiagen). cDNA was prepared from RNA using SuperScript III First-Strand Synthesis System (Thermo Fisher Scientific). The following primers were used to amplify zebrafish *snpha* and *snphb* cDNA: *snpha* forward, TGTCCTTCTGCATCCATGTC; *snpha* reverse, TCAGATAGGTGTCGCTCTTTC; *snphb* forward, ATGTCTTCGCCTTCAAATAAAAG; and *snphb* reverse, TCATATATTCATTCCCCCTGG. Amplicons were subcloned into Invitrogen PCRII-TOPO (Thermo Fisher Scientific), and were sequenced to verify gene identity and confirm sequence orientation for the generation of sense and antisense RNA probes.

### DNA plasmid constructs

All expression vectors were constructed using the Tol2kit Gateway cloning system (Kwan et al., 2007). We generated a pME-mitoEGFP entry clone by adding the mitochondrial targeting signal of the zebrafish *cox8a* gene to the 5´ end of EGFP sequence using a BP-compatible forward primer. We generated a pME-Lifeact-TagRFP entry clone by fusing the Lifeact sequence (Riedl et al., 2008) upstream of TagRFP using a BP-compatible forward primer. Lifeact-TagRFP was used to label F-actin in retinal axons and improve the visualization of growth cone filopodia. We generated a pME-TagBFP-*snphb* by adding the coding sequences of TagBFP (Evrogen) and a linker peptide (SGLRSRV) to the 5´ end of *snphb*. The p3E-2A-TagRFPCAAX-pA entry clone that encodes a 2A peptide (Provost et al., 2007) and TagRFP targeted to the plasma membrane by the prenylation motif of Ras (Moriyoshi et al., 1996) was a gift from Dr. Kristen Kwan (University of Utah, Salt Lake City, UT). The p5E-*isl2b* plasmid that drives specific expression in retinal ganglion cells (RGCs) was described previously (Pittman et al., 2008). Final isl2b:mitoEGFP-2A-TagRFPCAAX, isl2b:Lifeact-TagRFP, and isl2b:TagBFP-*snphb* plasmids were generated using LR reactions with the pDestTol2pA2 backbone (Kwan et al., 2007).

### RNA isolation and reverse transcriptase-coupled droplet digital PCR

For RNA isolation from whole embryos, 15 dechorionated WT embryos were lysed and homogenized in 500 μl TRIzol at cleavage, pharyngula prim-5 (24 hpf), long-pec (48 hpf), protruding mouth (72 hpf), larval day 4 (96 hpf), and larval day 5 (120 hpf). For RNA isolation from eyes at 72 hpf, 50 eyes were manually dissected from WT or *lak* mutant embryos. Total RNA was isolated using the Direct-zol RNA Miniprep Kit (Zymo Research) and eluted into 20 μl of nuclease-free water. cDNA was synthesized with 5 μg of purified RNA as an input using the SuperScript III System. Digital droplet PCR (ddPCR) was then performed on a QX200 AutoDG Instrument (Bio-Rad) using predesigned TaqMan primers and probes for zebrafish *snpha*, *sphnb*, *gapdh*, and *18s* (Table 1). Briefly, ddPCR reactions were assembled using Bio-Rad 2× ddPCR Supermix for Probes (no dUTP) and contained 1 μg of cDNA and 250 nm primers/probe. PCR amplification was performed with the following parameters: 39 cycles of 94°C for 30 s and 60°C for 1 min.

**Table 1:** Primers and probes used for ddPCR

Gene	Forward primer	Reverse primer	TaqMan probe	Amplicon size
*snpha*	GCAGCAGTTACTCAGCATCA	TGCCATGATTCTCACCACAG	TCCTGCAAGTGCACAGAGAGCATT	117
*snphb*	CACCTGTCAGTAACCGTGAT	TATGTGACGCCTATGGGTTG	AGCAGCAGTAGCAATTCAGGGTCA	107
*gapdh*	CCAAGGCTGTAGGCAAAGTA	GACTGTCAGATCCACAACAGAG	ACACGGAAGGCCATACCAGTAAGC	101
*18s*	GCCGCTAGAGGTGAAATTCT	TCGGAACTACGACGGTATCT	CAAGACGGACGAAAGCGAAAGCAT	129

### *In situ* hybridization

*In vitro* transcription of digoxigenin-labeled probes was performed using the RNA Labeling Kit (Roche Diagnostics) according to manufacturer instructions. Embryos were dechorionated at the appropriate developmental stages and fixed in 4% paraformaldehyde in PBS, pH 7.4, for 2 h at room temperature and overnight at 4˚C. Whole-mount *in situ* hybridization (ISH) was performed as described previously (Thisse and Thisse, 2008). Sense probes were used as controls. After staining, embryos were cleared in 80% glycerol. Images were acquired using an Olympus SZX16 stereomicroscope equipped with an Olympus DP80 dual color camera and Cellsens standard software.

### Imaging of mitochondria in retinal axons

Both isl2b:mitoEGFP-2A-TagRFPCAAX (30 pg) and isl2b:LifeAct-TagRFP (10 pg) plasmids were injected together with transposase mRNA into one-cell stage WT or *snph db* embryos. At 30–32 hpf, embryos were sorted for fluorescence, anesthetized in 0.015% tricaine, and embedded in a lateral view in 1% low-melt agarose in E3 medium + PTU. Their right eye was removed using a pulled glass pipette with a short taper as described in previous studies (Poulain et al., 2010; Poulain and Chien, 2013; Gaynes et al., 2015). Of note, removal of the contralateral eye preserves the underlying neuroepithelium, which ensures that the optic tract environment is not changed by the surgery and that retinal axon elongation and guidance are not affected. Embryos were allowed to recover until 46 hpf, when they were reanesthetized and about one-third of the yolk was removed by squeezing it out through a small hole torn with sharpened tungsten needles. Embryos were allowed to recover, reanesthetized at 50–52 hpf, and remounted in a lateral view in 1% low-melt agarose in E3 medium + PTU + tricaine in a membrane-bottomed Petri dish for time-lapse imaging on a Leica TCS SP8X laser-scanning confocal microscope equipped with LAS X software, HyD detectors, and a 40× objective (digital zoom 3, pinhole 1.25). *Z*-series were acquired for up to 3 h with 512 × 512 pixel resolution at 1 min intervals to minimize photobleaching. *Z*-intervals were 1 μm with a *z*-range of ∼35–40 μm to account for potential movement of the embryo. Chamber temperature was maintained at 28.5°C. Maximal intensity projections for each time point were compiled and aligned using ImageJ software (Schindelin et al., 2012; Schneider et al., 2012; RRID: SCR_002285) and the StackReg plugin (Thévenaz et al., 1998). Kymograph analyses of mitochondrial movement were performed using the ImageJ plugins KymoToolBox (Zala et al., 2013; RRID:SCR_016098) and KymoAnalyzer (Neumann et al., 2017). The number of mitochondria arriving at and leaving the growth cone were counted manually from the time-lapse sequences.

For the visualization of Snphb at the growth cone, isl2b:mitoEGFP-2A-TagRFPCAAX (15 pg), isl2b:LifeAct-TagRFP (10 pg), and isl2b:TagBFP-*snphb* (25 pg) plasmids were coinjected with transposase mRNA at the one-cell stage. Embryos were prepared in the same manner as for time-lapse imaging (described above). Single *z*-stacks (*z*-interval of 0.42 μm) were acquired at ∼50–52 hpf using a 40× objective with digital zoom 4, pinhole 1.0, and 1024 × 1024 pixel resolution.

For the visualization of mitochondria in single mature retinal axons at 120 hpf, embryos were injected with isl2b:mitoEGFP-2A-TagRFPCAAX (15 pg) and transposase mRNA at the one-cell stage and were selected for fluorescence in the optic tract at 96 hpf, after which the right eye was removed as described above. Larvae were reanesthetized at 120 hpf and mounted in a lateral view in 1.5% low-melt agarose in E3 medium + PTU + tricaine in a membrane-bottomed Petri dish. Imaging was performed using a 40× objective with digital zoom 2.5 and pinhole 1.0. Time-lapse recordings were performed in 35 *z*-planes (0.80 μm *z*-interval), allowing acquisition at 1 frame every 15 s over 10 min.

### Time-lapse image analysis

**Figure 1.**
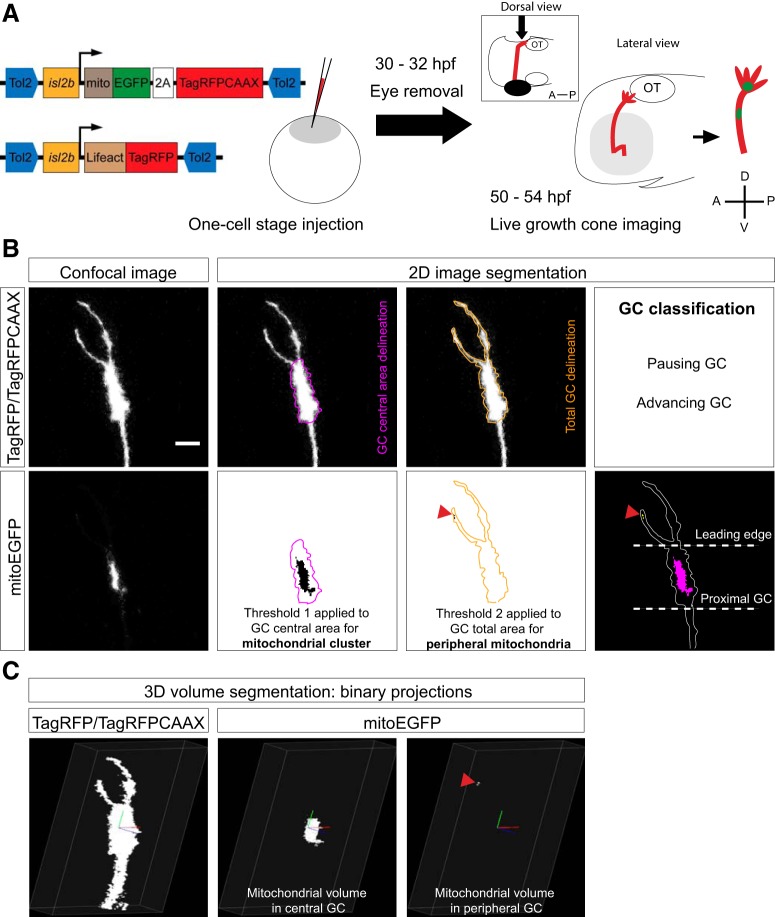
Live imaging approach and analysis. ***A***, Individual retinal axons and mitochondria were mosaically labeled by coinjecting isl2b:mitoEGFP-2A-TagRFPCAAX and isl2b:Lifeact-TagRFP plasmids at the one-cell stage. isl2b:Lifeact-TagRFP was used to improve the visualization of single axons and growth cone (GC) filopodia (for more details, see main text and Materials and Methods). After removal of the contralateral eye, axons growing along the optic tract toward the optic tectum (OT) and their mitochondria were imaged in a lateral view between 50 and 54 hpf (Δ*t* = 1 min). A, Anterior; D, dorsal; OT, optic tectum; P, posterior; V, ventral. ***B***, Growth cone total and central areas visualized with TagRFP/TagRFPCAAX were manually segmented and used as ROIs for segmenting mitochondrial signals using the particle analysis tool in ImageJ. Two different thresholds were used to analyze mitochondrial particles: a threshold of 55 was applied to the growth cone central area to segment the main mitochondrial cluster, and a threshold of 20 was applied to the growth cone total area to segment smaller individual mitochondria present in the peripheral area (red arrowheads). Time-lapse recordings were classified according to growth cone behavior. Growth cone leading edge and proximal growth cone are indicated in the merged image; see Materials and Methods for definitions. Lateral view, confocal maximal projections. Scale bar, 5 μm. ***C***, Volumes of mitochondria and GC total and central volumes were calculated using 3D analysis. GC total and central volumes visualized with TagRFP/TagRFPCAAX were calculated on binary *z*-projections and used as volumes of interest for segmenting mitochondrial volumes using the voxel counter plugin in ImageJ (see also Movie 1).

Image analysis was conducted using ImageJ (Schindelin et al., 2012; Schneider et al., 2012). For 2D analyses, the TagRFP/TagRFPCAAX signals from stack maximal projections were used to manually segment the growth cone total and central areas. The growth cone central area was determined by tracing the perimeter of the growth cone body, not including filopodia. As such, the central area is an arbitrary outline based on growth cone morphology and fluorescent intensity that presumably corresponds to growth cone central domain and transition zone (it does not necessarily correspond to what is commonly defined as the growth cone central domain based on cytoskeletal components). Regions of interest (ROIs) defined as the total and central areas were used to segment the mitoEGFP signals on binary images using the “particle analysis” tool. The following two different thresholds were used to analyze mitochondrial particles: a threshold of 55 was applied to the growth cone central area to segment the main mitochondrial cluster; and a threshold of 20 was applied to the growth cone total area to segment smaller individual mitochondria present in the peripheral area (Fig. 1*B*). A threshold of 20 was also applied to analyze pioneering mitochondrial clusters using the particle analysis tool. The number, area, and presence (percentage of time of advance) of pioneering mitochondrial clusters were analyzed in every frame of an advancing growth cone central area. The peripheral area, comprising filopodia, was calculated by subtracting the growth cone central area from the growth cone total area. The number of filopodia was counted manually. We drew a straight line from the mitochondrial cluster boundary to the most distal outline of the growth cone central area where two filopodia join together to measure the distance between the main mitochondrial cluster and the growth cone leading edge. We also defined the proximal growth cone as the most proximal boundary of the growth cone central area, where the growth cone transitions into the axon shaft. A growth cone was classified as advancing when the proximal growth cone advanced >1 μm after an elongation of the growth cone central area (i.e. major >3× minor axis length of the best fitting ellipse of the central area). Elongation rates were quantified by measuring the displacement between the proximal growth cone boundaries before and after advance and expressed in micrometers per minute.

Visualizations of 3D growth cones and mitochondria were prepared using FluoRender (RRID:SCR_014303; Wan et al., 2017). For volumetric analysis of mitochondrial occupancy at the growth cone, *z*-stacks were processed and analyzed with ImageJ. Images were thresholded to the lowest level that excludes the majority of noise pixels to obtain binary *z*-projections similar to raw *z*-projections, after which volumes were calculated using the Voxel Counter plugin. Volume data of mitochondria and the growth cone, expressed in cubic micrometers, were divided to obtain percentages of occupancy. Figures were prepared using Adobe Photoshop and Illustrator, and time-lapse videos were assembled using ImageJ.

### Statistics

Data were analyzed and graphs were produced using Prism (GraphPad Software). Data are presented as the mean ± SEM. Statistical tests were applied as indicated in the Results and figure legends. Additional statistical details are provided in Table 2. Normal distribution was determined by column test.

**Table 2:** Summary of statistical analyses

Figure	Measurement	Data structure	Type of test	Comparison	Statistical value
Fig. 2*B*	Mitochondrial occupancy (GC total volume)	Normal	Unpaired *t* test	Pausing vs advancing GC	*p* = 0.4065 *t*_(17)_ = 0.8511
Fig. 2*B’*	Mitochondrial occupancy (GC total area)	Normal	Unpaired *t* test	Pausing vs advancing GC	*p* = 0.4310 *t*_(17)_ = 0.8067
Fig. 2*C*	Mitochondrial occupancy (GC central volume)	Normal	Unpaired *t* test	Pausing vs advancing GC	*p* = 0.2829 *t*_(17)_ = 1.109
Fig. 2*C’*	Mitochondrial occupancy (GC central area)	Normal	Unpaired *t* test	Pausing vs advancing GC	*p* = 0.2325 *t*_(17)_ = 1.238
Fig. 2*D*	Mitochondrial volume (peripheral volume)	Normal	Unpaired *t* test	Pausing vs advancing GC	*p* = 0.4444 *t*_(17)_ = 0.7831
Fig. 2*D’*	Number of mitochondria in GC peripheral area	Normal	Unpaired *t* test	Pausing vs advancing GC	*p* = 0.7028 *t*_(15)_ = 0.3889
Fig. 2*E*	Distance from leading edge	Normal	Unpaired *t* test	Pausing vs advancing GC	*p* < 0.0001 *t*_(17)_ = 6.740
Fig. 4*B*	Percentage net mitochondrial transport	Normal	Two-way ANOVA *(post hoc* Bonferroni)	Pausing vs advancing GC	*p* = 0.9955 *F*_(1,34)_ = 0.00003
Fig. 4*C*	Percentage time mitochondria spent mobile	Normal	Unpaired *t* test	Pausing vs advancing GC	*p* = 0.0131 *t*_(17)_ = 2.772
Fig. 4*C*	Percentage time mitochondria spent stationary	Normal	Unpaired *t* test	Pausing vs advancing GC	*p* = 0.0075 *t*_(17)_ = 3.034
Fig. 4*D*	Mitochondrial flux (arriving mitochondria)	Normal	Unpaired *t* test	Pausing vs advancing GC	*p* = 0.4666 *t*_(17)_ = 0.7448
Fig. 4*D*	Mitochondrial flux (leaving mitochondria)	Normal	Unpaired *t* test	Pausing vs advancing GC	*p* = 0.3617 *t*_(17)_ = 0.9374
Fig. 4*E*	Number of arriving mitochondria vs axon growth	Normal	Linear regression		*p* = 0.0071 *r*² = 0.5324
Fig. 5*D*	*snpha* expression	Normal	Unpaired *t* test	WT vs *lak*	*p* < 0.0001 t_(4)_ = 18.33
Fig. 5*D*	*snphb* expression	Normal	Unpaired *t* test	WT vs *lak*	*p* < 0.0001 *t*_(4)_ = 17.84
Fig. 6*E*	Percentage stationary mitochondria	Normal	Unpaired *t* test	WT vs *snph db*	*p* = 0.0074 *t*_(13)_ = 3.168
Fig. 7*A*	GC total occupancy (percentage volume)	Normal	Unpaired *t* test	WT vs *snph db*	*p* = 0.0023 *t*_(22)_ = 3.446
Fig. 7*A’*	GC total occupancy percentage area)	Normal	Unpaired *t* test	WT vs *snph db*	*p* = 0.0178 *t*_(22)_ = 2.562
Fig. 7*B*	GC central occupancy (percentage volume)	Normal	Unpaired *t* test	WT vs *snph db*	*p* = 0.0184 *t*_(22)_ = 2.543
Fig. 7*B’*	GC central occupancy (percentage area)	Normal	Unpaired *t* test	WT vs *snph db*	*p* = 0.0071 *t*_(22)_ = 2.967
Fig. 7*C*	Peripheral mitochondrial volume	Normal	Unpaired *t* test	WT vs *snph db*	*p* = 0.2765 *t*_(22)_ = 1.116
Fig. 7*C’*	Number of mitochondria in peripheral area	Normal	Unpaired *t* test	WT vs *snph db*	*p* = 0.6030 *t*_(21)_ = 0.5278
Fig. 7*D*	Distance from leading edge (pausing GC)	Normal	Unpaired *t* test	WT vs *snph db*	*p* = 0.0003 *t*_(20)_ = 4.403
Fig. 7*D’*	Distance from leading edge (advancing GC)	Normal	Unpaired *t* test	WT vs *snph db*	*p* = 0.1080 *t*_(16)_ = 1.703
Fig. 7*E*	Mitochondrial flux (arriving mitochondria)	Normal	Unpaired *t* test	WT vs *snph db*	*p* = 0.4976 *t*_(22)_ = 0.6904
Fig. 7*E*	Mitochondrial flux (leaving mitochondria)	Normal	Unpaired *t* test	WT vs *snph db*	*p* = 0.0412 *t*_(22)_ = 2.168
Fig. 7*F*	Percentage net mitochondrial transport (anterograde)	Normal	Unpaired *t* test	WT vs *snph db*	*p* = 0.0178 *t*_(22)_ = 2.561
Fig. 7*F*	Percentage NET mitochondrial transport (retrograde)	Normal	Unpaired *t* test	WT vs *snph db*	*p* = 0.0529 *t*_(22)_ = 2.046
Fig. 7*G*	Percentage time mitochondria spent in motion (anterograde)	Normal	Unpaired *t* test	WT vs *snph db*	*p* = 0.3330 *t*_(22)_ = 0.9900
Fig. 7*G*	Percentage time mitochondria spent in motion (retrograde)	Normal	Unpaired *t* test	WT vs *snph db*	*p* = 0.5596 *t*_(22)_ = 0.5924
Fig. 7*H*	GC total area	Normal	Unpaired *t* test	WT vs *snph db*	*p* = 0.2904 *t*_(22)_ = 1.083
Fig. 7*H*	GC central area	Normal	Unpaired *t* test	WT vs *snph db*	*p* = 0.3869 *t*_(22)_ = 0.8829
Fig. 7*I*	Number of filopodia	Normal	Unpaired *t* test	WT vs *snph db*	*p* = 0.6775 *t*_(22)_ = 0.42154
Fig. 7*J*	Growth rate	Normal	Unpaired *t* test	WT vs *snph db*	*p* = 0.8256 *t*_(15)_ = 0.2243
Fig. 8*D*	Pioneering cluster area	Normal	Unpaired *t* test	WT vs *snph db*	*p* = 0.0166 *t*_(15)_ = 2.697
Fig. 8*E*	Number of pioneering clusters	Normal	Unpaired *t* test	WT vs *snph db*	*p* = 0.1865 *t*_(15)_ = 1.385
Fig. 8*F*	Mitochondria presence (% time)	Normal	Unpaired *t* test	WT vs *snph db*	*p* = 0.1994 *t*_(15)_ = 1.343

## Results

### Mitochondrial localization at the growth cone correlates with axon outgrowth

**Figure 2.**
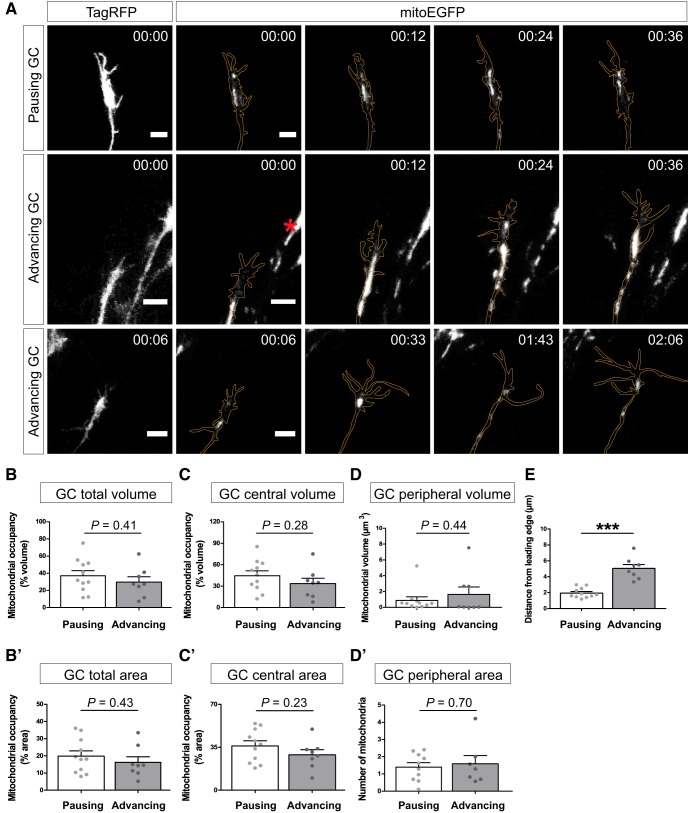
Mitochondrial distribution changes with growth cone behavior. ***A***, Representative time-lapse images of mitochondria (mitoEGFP, white) in distal retinal axons and growth cones (labeled with TagRFP, delineated in orange and shown in left panels) classified as pausing or advancing (see Movies 2, 3, 4). Most growth cones alternate periods of pausing and advancing/elongating, resulting in net axon growth, as shown in the bottom panels. The red asterisk indicates mitochondria in neighboring axons. Scale bar, 5 μm. ***B*–*C*´**, Quantification of the mitochondrial occupancy (as a percentage) of the growth cone total volume (***B***), total area (***B´***), central volume (***C***), and central area (***C´***). ***D***, ***D´***, Quantification of the mitochondrial volume in the peripheral growth cone (***D***) and of the number of mitochondria in the growth cone peripheral area (***D´***). ***E***, Quantification of the distance between the largest mitochondrial cluster and the growth cone leading edge. When growth cones elongate, the distance to the growth cone leading edge increases significantly. Data from 12 independent experiments (pausing, *n* = 11; advancing, *n* = 8) are shown as mean ± SEM. Statistical analysis (***B–E***): unpaired *t* test, ***p < 0.001.

To monitor mitochondrial behavior in developing axons *in vivo*, we performed high-resolution confocal time-lapse imaging of mitochondria in retinal axons elongating along the optic tract of zebrafish embryos between 50 and 55 hpf (Fig. 1*A*). We coexpressed EGFP targeted to mitochondria (mitoEGFP) and TagRFP targeted to the plasma membrane (TagRFPCAAX), and to actin filaments (Lifeact-TagRFP) in single retinal axons, and monitored labeled axons elongating along the contralateral optic tract toward the optic tectum (Fig. 1, Movie 1). As described previously (Bovolenta and Mason, 1987; Holt and Harris, 1993), growth cones of retinal axons in the tract were slender with numerous filopodia protruding and retracting dynamically. We could distinguish two classes of axons depending on their outgrowth status (Fig. 2*A*). Advancing axons had very elongated growth cones, whereas pausing, not growing, axons had rounder growth cones with filopodia oriented in all directions, supporting the notion that growth cone shape correlates with growth cone behavior (Bovolenta and Mason, 1987; Mason and Wang, 1997). For the duration of our time-lapse videos (range, 30–160 min), several growth cones were found to alternate between pausing and advancing behaviors and could therefore be classified into both categories (Movies 2, 3, 4).

Movie 1. Representative 3D visualization of mitochondria in a pausing growth cone. Video corresponding to Figure 1*C* showing mitochondria (green) in a distal retinal axon and growth cone (red) pausing along the optic tract. Scale bar, 5 μm. 10.1523/ENEURO.0026-19.2019.video.1

Movie 2. Representative time-lapse recording of mitochondrial dynamics in an advancing growth cone. Time-lapse video corresponding to Figure 2*A* showing mitochondria (green, white) in a distal retinal axon and growth cone (red) elongating along the optic tract. Images were acquired at 1 min intervals for 48 min. Each frame is a confocal image stack maximal projection, lateral view, and anterior is on the left. Time stamp format: hours:minutes (hr:min). Scale bar, 5 μm. 10.1523/ENEURO.0026-19.2019.video.2

**Figure 3.**
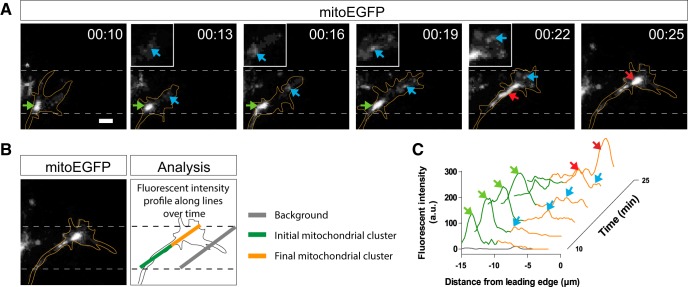
Pioneering mitochondrial clusters localize near the leading edge in advancing growth cones. ***A***, Representative time-lapse images of mitochondria (mitoEGFP, white) in an advancing growth cone (Movie 5). While the main mitochondrial cluster (green arrow) lags behind during growth cone advance, some small pioneering mitochondrial clusters (blue arrows) appear in close proximity to the leading edge. Lateral view, confocal maximal projections. Scale bar, 3 μm. ***B***, ***C***, EGFP intensity profiles calculated along a line between the initial proximal growth cone and final leading edge to analyze the distribution of mitochondria along the advancing growth cone at successive time points shown in ***A***. ***C***, The orange and green lines correspond to the fluorescent intensities of mitochondria in the growth cone and at the proximal growth cone, respectively (see ***B***: Analysis).

Movie 3. A subset of mitochondria localizes to the leading edge of the growth cone during elongation. Representative time-lapse video corresponding to Figure 3*A* showing mitochondria (green, white) in a distal retinal axon and growth cone (red) elongating along the optic tract. Images were acquired at 1 min intervals for 36 min. Note that the growth cone is elongating from 00:10 to 00:25. During this elongation, most mitochondria lag behind, but a subset of smaller mitochondria localizes adjacent to the leading edge. Each frame is a confocal image stack maximal projection, lateral view, and anterior is on the left. Time stamp format: hours:minutes (hr:min). Scale bar, 3 μm. 10.1523/ENEURO.0026-19.2019.video.3

Movie 4. Representative time-lapse recording of mitochondrial dynamics in a pausing growth cone. Time-lapse video corresponding to Figure 2*A* showing mitochondria (green, white) in a distal retinal axon and growth cone (red) pausing along the optic tract. Images were acquired at 1 min intervals for 48 min. Each frame is a confocal image stack maximal projection, lateral view, and anterior is on the left. Time stamp format: hours:minutes (hr:min). Scale bar, 5 μm. 10.1523/ENEURO.0026-19.2019.video.4

As previously observed *in vitro* (Morris and Hollenbeck, 1993), mitochondria were abundantly detected at the growth cone (Fig. 2*A*, Movies 1, 2, 3, 4). The majority of mitochondria clustered in the microtubule-rich growth cone central area, whereas smaller mitochondria regularly appeared in the peripheral area along protrusions or actin filopodia (Movie 3). We analyzed mitochondrial localization and distribution at the growth cone by quantifying mitochondrial occupancy in the growth cone total and central volumes (Fig. 2*B*,*C*), peripheral mitochondrial volume (Fig. 2*D*), mitochondrial occupancy in the growth cone total and central areas (Fig. 2*B*´,*C*´), the number of mitochondria in the peripheral area (Fig. 2*D*´), and the distance between mitochondria and the growth cone leading edge (Fig. 2*E*; see also Fig. 1*B*,*C*). Mitochondrial occupancy was chosen as a measure to evaluate mitochondrial density in the growth cone since spatial overlap of mitochondria within the growth cone central zone did not allow the visualization and quantification of single mitochondria. Mitochondrial occupancy in the total and central volumes and areas were similar in advancing versus pausing growth cones (Fig. 2*B*–*C*´). We did note that when we exclusively compared growth cones that frequently alternated between pausing and advancing behaviors, a significant decrease in mitochondrial occupancy in the central area of advancing growth cones was observed (paired *t* test, *p* = 0.006, *t*_(6)_ = 4.2). Furthermore, the distance of the main mitochondrial cluster to the leading edge was significantly different between pausing and advancing growth cones (Fig. 2*E*). When a growth cone advanced, its central area elongated substantially, but this forward extension was not accompanied with a forward advance of mitochondria (Fig. 3*A*, Movie 5). Hence, the distance between them and the leading edge increased twofold (Fig. 2*E*). Mitochondria repositioned near the leading edge when the growth cone regained a rounder shape (Fig. 3*A*), which often indicated a transition to a pausing state. Interestingly, although the majority of mitochondria lagged behind during growth cone extension, a smaller subset of mitochondria was always detected adjacent to the leading edge (Fig. 3, Movie 5). We analyzed the number and area of these “pioneering mitochondrial clusters,” as well as the amount of time they were present when a growth cone advanced. We found that pioneering mitochondrial clusters had on average a total area of 1.1 ± 0.4 μm^2^, and that 2.6 ± 0.3 mitochondrial clusters positioned near the leading edge in 43 ± 9.5% of the time of advance. Importantly, pioneering mitochondria were consistently observed in every growth cone that elongated.

Movie 5. Representative time-lapse recording of mitochondrial dynamics in a growth cone that combines pausing and advancing. Time-lapse video corresponding to Figure 2*A* showing mitochondria (green, white) in a distal retinal axon and growth cone (red) that alternates between pausing and elongating behaviors. The growth cone is pausing during the majority of the recording, but advances intermittently (e.g., from 00:00 to 00:18, 01:39 to 01:55, and 02:24 to 02:39). Images were acquired at 1 min intervals for 160 min. Each frame is a confocal image stack maximal projection, lateral view, and anterior is on the left. Time stamp format: hours:minutes (hr:min). Scale bar, 5 μm. 10.1523/ENEURO.0026-19.2019.video.5

Altogether, our data show that mitochondria localize in the growth cone central area *in vivo* and are also present along filopodia extending from the growth cone periphery. The position of mitochondria in the growth cone central area further changes with the growth status of the axon.

### Mitochondrial transport is coordinated with growth cone behavior and axon growth *in vivo*

**Figure 4.**
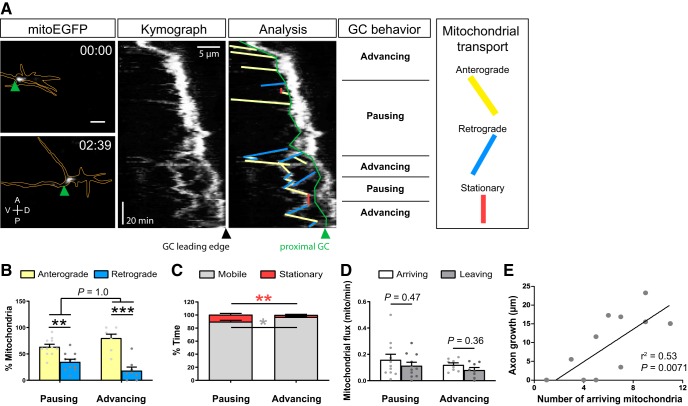
Anterograde mitochondrial transport correlates with axonal outgrowth. ***A***, Representative kymograph of mitochondria (mitoEGFP, white) in a distal axon whose growth cone alternates between advancing and pausing. The first and last frames of the time-lapse recording (Movie 4) are shown with axon and growth cone delineated in orange. Confocal maximal projections. Scale bar, 5 μm. The growth cone leading edge and proximal growth cone (green line) are indicated on the kymograph and kymograph analysis panels. ***B***, Quantification of net transport, analyzed by counting the number of mitochondria moving anterogradely or retrogradely. Data from 12 independent experiments (pausing: *n* = 11, advancing: *n* = 8) are shown as the mean ± SEM. Statistical analysis: two-way ANOVA with *post hoc* Bonferroni test, **p < 0.01, ***p < 0.001. ***C***, Quantification of the percentage of time mitochondria spent in a mobile or stationary state. A significant increase in time spent in motion (grey asterisk, *p* = 0.013) and a decrease in time spent in a stationary state (red asterisk, *p* = 0.008) are observed proximally to growth cones that are advancing. Data from 12 independent experiments (pausing, *n* = 11; advancing, *n* = 8) are shown as the mean ± SEM. Statistical analysis: unpaired *t* test. ***D***, Quantification of mitochondrial flux, showing a trend toward more mitochondria arriving versus leaving the growth cone in both pausing and advancing growth cones. Data from 12 independent experiments (pausing, *n* = 11; advancing, *n* = 8) are shown as the mean ± SEM. Statistical analysis: unpaired *t* test. ***E***, Linear regression analysis between the number of mitochondria arriving at a growth cone and growth cone advance (*n* = 12).

Next, we asked whether transport of mitochondria in the axon shaft proximal to the growth cone also relates to growth status. We performed kymograph analysis to measure net mitochondrial transport as well as the percentage of time mitochondria spent in motion (Fig. 4*A–C*). In agreement with a higher mobility of mitochondria observed in developing versus mature axons (Lewis et al., 2016), <5% of all mitochondria were stationary (Fig. 4*C*). The majority of mobile mitochondria moved anterogradely, with a significantly higher percentage of anterograde versus retrograde trafficking in both pausing and advancing growth cones (Fig. 4*B*). An analysis of mitochondrial transport over shorter timescales showed a similar proportion of mitochondria moving anterogradely (71 ± 5.4% in long movies, 78 ± 7.3% over shorter timescales), retrogradely (26 ± 4.5% in long movies, 20 ± 6.7% over shorter timescales), or remaining stationary (2.3 ± 1.6% in long movies, 2.3 ± 2.3% over shorter timescales), indicating that the length of time-lapse movies had no effect on mitochondrial transport parameters (two-way ANOVA, effect of time, *p* = 0.9994, *F*_(1.66)_ < 0.0001). Mitochondria spent more time moving when a growth cone advanced (Fig. 4*C*), and we observed a trend toward more mitochondria arriving versus leaving in both pausing and advancing growth cones (Fig. 4*D*). We finally found a strong correlation between the number of mitochondria arriving at the growth cone and the distance the axon elongated (Fig. 4*E*). Altogether, these results illustrate that mitochondrial transport toward the growth cone correlates with growth cone advance, suggesting that mitochondrial motility and axon elongation may be functionally linked or coregulated *in vivo*.

### Zebrafish Syntaphilins are expressed in RGCs and localize to the growth cone

**Figure 5.**
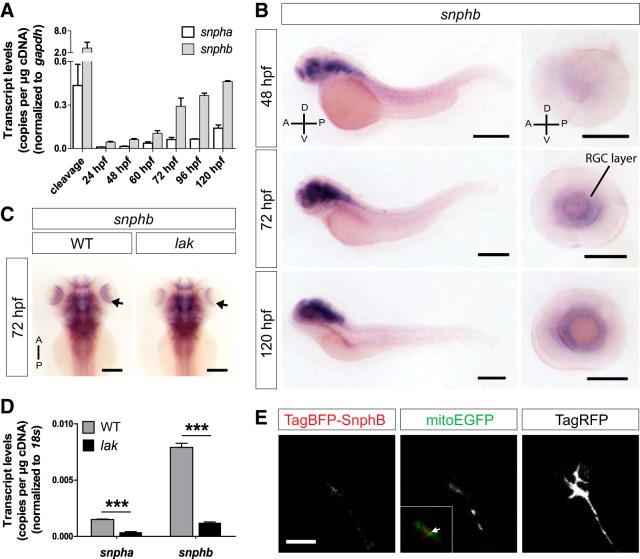
Zebrafish Syntaphilins are expressed in RGCs during development. ***A***, Quantification of *snpha* and *snphb* mRNA levels during embryonic development by RT-ddPCR. mRNA levels were normalized to that of *gapdh* used as a control. Data from three independent experiments are shown as the mean ± SEM. ***B***, Lateral views of whole embryos stained for *snphb* by ISH show predominant expression in the brain at 48, 72, and 120 hpf. *snphb* is also increasingly expressed in the RGC layer over time. Scale bars: whole embryos, 400 μm; eyes, 200 μm. ***C***, Dorsal views of WT and RGC-deficient *lak* mutant embryos stained for *snphb* by ISH at 72 hpf. Expression of *snphb* is decreased in the retinae of *lak* embryos (arrows). Scale bar, 200 μm. ***D***, Quantification of *snpha* and *snphb* mRNA levels in the eyes of WT and *lak* embryos at 72 hpf analyzed by RT-ddPCR. Transcript levels were normalized to that of *18s*, which was used as a control. Data from three experiments are shown as the mean ± SEM. Statistical analysis: unpaired *t* test, ***p < 0.001. ***E***, TagBFP-Snphb localizes to the growth cone of elongating axons *in vivo*. Isl2b:TagBFP-*snphb,* isl2b:mitoEGFP-2A-TagRFPCAAX, and isl2b:Lifeact-TagRFP were coexpressed in individual RGCs. TagBFP-Snphb and mitochondria are both present in the growth cone (arrow in merged image). Lateral view, confocal maximal projections. Scale bar, 5 μm.

**Figure 6.**
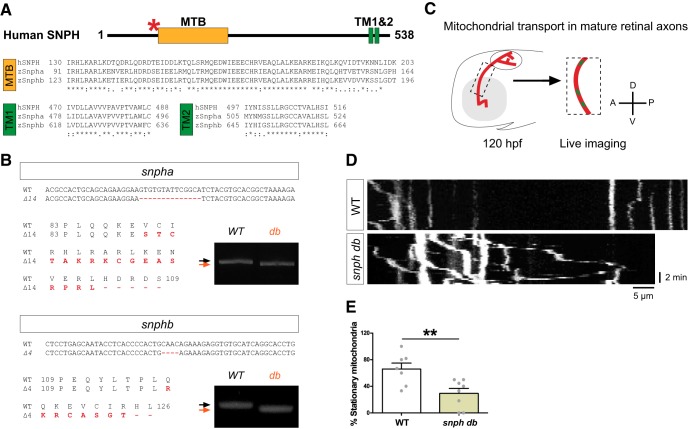
Zebrafish Syntaphilins anchor mitochondria in mature retinal axons. ***A***, Domain structure of human SNPH. Both the MTB and TMs are highly conserved in zebrafish Snpha and Snphb (see also Extended Data Figure 6-1). The red asterisk indicates the position of the TALEN target region. ***B***, Mutations in *snpha* and *snphb* were introduced by TALEN mutagenesis. Red lines indicate the deleted sequences. Changes in amino acids are shown in red. RT-PCR analysis of *snpha* and *snphb* in WT and *db* embryos demonstrate the presence of shorter transcripts in the mutants. ***C***, Individual retinal axons and mitochondria were mosaically labeled by injecting isl2b:mitoEGFP-2A-TagRFPCAAX at the one-cell stage. After removal of the contralateral eye, mature axons of the optic tract and their mitochondria were imaged in a lateral view at 120 hpf (Δ*t* = 15 s). ***D***, ***E***, Quantification of the percentage of stationary mitochondria using kymograph analysis shows a reduction in stalled mitochondria in axons from *snph db* mutants. Data from two independent experiments (WT: *n* = 7, *db*: *n* = 8) are shown as the mean ± SEM. Statistical analysis: unpaired *t* test, **p < 0.01.

How mitochondria are maintained in the growth cone remains poorly understood. One possible mechanism involves the attachment, or docking, of mitochondria to microtubules by Snph. Snph was identified as an outer mitochondrial membrane protein that is able to dock mitochondria directly to microtubules via its microtubule-binding domain (MTB; Kang et al., 2008; see Fig. 6*A*). Snph was shown to be important in axons for the regulation of branching (Courchet et al., 2013) and synaptic plasticity (Kang et al., 2008). We decided to test whether Snph also plays a role in docking mitochondria at the growth cone during axon elongation *in vivo*. Due to the whole-genome duplication that occurred in the teleost lineage (Glasauer and Neuhauss, 2014), two *snph* genes, *snpha* and *snphb*, are present in zebrafish. Both Snpha and Snphb share a high sequence conservation with human SNPH in their MTB and mitochondrial transmembrane domains (TMs), strongly suggesting a conserved mitochondrial anchoring function (see Fig. 6*A*, Extended Data Fig. 6-1). We first analyzed the expression of *snpha* and *snphb* during zebrafish development. Quantification of *snpha* and *snphb* mRNA levels using reverse transcriptase (RT)-coupled ddPCR showed that both *snpha* and *snphb* are maternally expressed and that mRNA levels for both *snphs* increase over later developmental periods, albeit at much lower levels for *snpha* (Fig. 5*A*). ISH further revealed a high expression of *snphb* in the nervous system at 48, 72, and 120 hpf (Fig. 5*B*), whereas *snpha* expression was not detectable using this approach (data not shown). Importantly, *snphb* was detected in the RGC layer at the time of retinal axon elongation (Fig. 5*B*), suggesting a possible role in anchoring mitochondria in retinal growth cones. To further test whether *snphb* is expressed by RGCs, we performed ISH on WT and RGC-deficient *lak* mutant embryos (Kay et al., 2001) at 72 hpf (Fig. 5*C*). *Snphb* expression was strongly reduced in the retina of *lak* compared with WT, while it appeared unchanged in the brain (Fig. 5*C*). This decreased retinal expression was further confirmed by RT-ddPCR performed on dissected eyes (Fig. 5*D*). *Snphb* mRNA levels were highly detected in WT but significantly reduced in *lak*, indicating that *snphb* is strongly expressed by RGCs. Interestingly, *snpha* transcripts were also detected in WT, albeit at much lower levels. Like *snphb*, *snpha* eye expression was significantly decreased in *lak* (Fig. 5*D*), indicating that both Snphs are present in RGCs at the time of axon elongation.

10.1523/ENEURO.0026-19.2019.f6-1 Figure 6-1 Zebrafish Syntaphilins have a highly conserved microtubule-binding domain and mitochondrial attachment sites. ***A***, Depiction of the domain structure of human SNPH and zebrafish Snpha and Snphb. The MTB and TMs are indicated in orange and green, respectively. ***B***, Sequence alignment of human SNPH and zebrafish Snpha and Snphb. The MTB (orange) and TMs (green) are highly conserved, suggesting a conserved function in anchoring mitochondria to microtubules. Download Figure 6-1, EPS file.

As *snphb* is highly expressed in RGCs, we finally tested whether Snphb could localize to the growth cones of retinal axons elongating along the tract. As we could not identify an antibody directed against mammalian Snph that would specifically recognize zebrafish Snphs (data not shown), we decided to coexpress TagBFP-tagged Snphb, mitoEGFP, TagRFPCAAX, and Lifeact-TagRFP in single retinal axons, and to monitor labeled growth cones advancing along the contralateral optic tract (Fig. 5*E*). We detected TagBFP-Snphb in the central area of retinal growth cones, where it appeared to colocalize with the main mitochondrial cluster. Altogether, our results show that Snphs are expressed by RGCs and can localize to the growth cone of developing axons, suggesting they might participate to mitochondria docking at the growth cone during axon elongation *in vivo.*

### Syntaphilins contribute to mitochondrial immobilization at the growth cone

To test the function of zebrafish Snphs in retinal axons, we generated *snpha* and *snphb* mutants using TALEN-mediated genome editing (Fig. 6*A*,*B*). We targeted a region upstream of the MTB to ensure full loss of function and obtained two alleles consisting of a 14 and 4 bp deletion for *snpha* and *snphb*, respectively, which were predicted to cause premature termination of translation (Fig. 6*B*). As both *snpha* and *sphnb* are expressed in RGCs (Fig. 5*D*) and to avoid potential compensation mechanisms, we generated maternal zygotic double mutants (*db*) and confirmed by RT-PCR and cDNA sequencing that both mutant mRNAs were expressed and contained the corresponding mutations (Fig. 6*B*). No alternative transcripts were detected in *db* (data not shown). *db* were viable and fertile, and *db* embryos did not show any obvious morphologic abnormalities, as was reported for *Snph* knock-out (KO) mice (Kang et al., 2008). To test whether zebrafish Snphs also contribute to mitochondrial anchoring in axons, we quantified mitochondrial transport in mature retinal axons of WT and *db* embryos at 120 hpf (Fig. 6*C*), when most mitochondria are known to become immobile (Lewis et al., 2016; Smit-Rigter et al., 2016). As expected, mitochondrial mobility was strongly increased in axons of *db* (Fig. 6*D*,*E*), demonstrating that the mitochondrial docking function of Snph is conserved in zebrafish.

**Figure 7.**
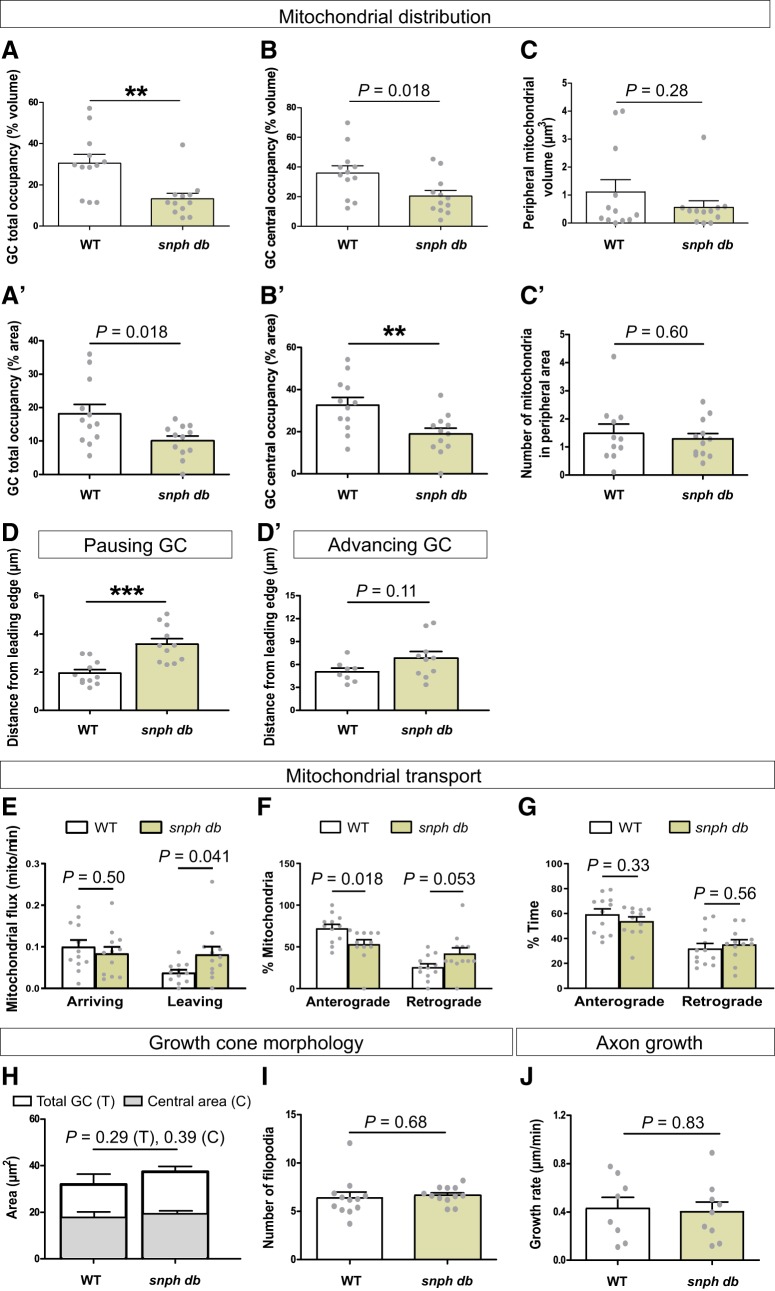
Syntaphilins participate in mitochondrial docking at the growth cone but do not regulate axon elongation. ***A–B´***, Quantification of the mitochondrial occupancy (as a percentage) of the growth cone total volume (***A***), total area (***Á***), central volume (***B***), and central area (***B´***) in WT and *snph db*. Mitochondrial occupancy is decreased in *snph db.* ***C***, ***C´***, Quantification of the mitochondrial volume in the peripheral growth cone (***C***), and of the number of mitochondria in the growth cone peripheral area (***C´***) in WT and *snph db*. ***D***, ***D´***, Quantification of the distance between the largest mitochondrial cluster and the growth cone leading edge in WT and *snph db*. We quantified the distance from the leading edge separately in pausing and advancing growth cones as it varies depending on growth cone status (Fig. 2*E*). Mitochondria are located further from the leading edge in retinal growth cones of *db* compared with WT. ***E–G***, Reduced mitochondrial occupancy in the growth cone of *snph db* is accompanied by an increased removal of mitochondria from the growth cone. More mitochondria leave the growth cone per minute in *db*, while no difference is detected for arriving mitochondria (***E***). Quantification of net mitochondrial transport proximally to the growth cone (***F***) shows more mitochondria moving retrogradely in *snph db*, which is accompanied by a decreased percentage of mitochondria moving in the anterograde direction. No differences in percentage time in anterograde or retrograde motion were found (***G***). ***H***, ***I***, Quantification of growth cone morphology shows no differences in growth cone total and central areas (***H***) and number of filopodia (***I***) between *db* and WT embryos. ***J***, Axon elongation is not statistically different between *db* and WT embryos. Data from 12 independent experiments per genotype are shown as the mean ± SEM. Statistical analysis: unpaired *t* test, **p < 0.01, ***p < 0.001.

We next performed time-lapse imaging of mitochondria in retinal axons elongating along the optic tract of *db* embryos to test whether Snphs also play a role in mitochondrial tethering at the growth cone of growing axons (Movies 6, 7, 8). We compared *db* with WT embryos that have a similar genetic background (datasets analyzed in Figs. 2–4). Interestingly, mitochondrial distribution at the growth cone was modified in *db* compared with WT (Fig. 7*A–D*´). While a same mitochondrial volume and number were observed in the growth cone peripheral volume and area in WT and *db* (Fig. 7*C*,*C*´), mitochondria occupied a smaller proportion of the growth cone total and central volumes and areas in *db* (Fig. 7*A*,*B*´, Movies 6, 7, 8). Moreover, mitochondria were located further from the growth cone leading edge in *db*, which was most pronounced in pausing growth cones (Fig. 7*D*,*D*´). Interestingly, the decreased mitochondrial occupancy of the growth cone in *db* was accompanied with changes in mitochondrial flux. While a similar number of mitochondria arrived at the growth cone, more mitochondria left the growth cone in *db* versus WT (Fig. 7*E*), suggesting that Snphs dock mitochondria once they have reached the growth cone. The increased departure of mitochondria from the growth cone of *db* was associated with changes in net mitochondrial transport proximally to the growth cone. More mitochondria moved retrogradely in *snph db*, which was accompanied by a decreased percentage of mitochondria moving in the anterograde direction (Fig. 7*F*). No differences were found between WT and *db* in the amount of time that mitochondria moved in both directions (Fig. 7*G*).

Movie 6. Representative 3D visualization of mitochondria in a pausing growth cone of a *snph db mutant* embryo. Video showing mitochondria (green) in a distal retinal axon and growth cone (red) pausing along the optic tract. Scale bar, 5 μm. 10.1523/ENEURO.0026-19.2019.video.6

Movie 7. Representative time-lapse recording of mitochondrial dynamics in a growth cone of a *snph db* mutant embryo. Time-lapse video showing mitochondria (green, white) in a distal retinal axon and growth cone (red) elongating along the optic tract of a *snph db* embryo. Images were acquired at 1 min intervals for 96 min. Note that the growth cone is combining periods of pausing with advancing (from 00:00 to 00:10 and 00:53 till end). Each frame is a confocal image stack maximal projection, lateral view, and anterior is on the left. Time stamp format: hours:minutes (hr:min). Scale bar, 5 μm. 10.1523/ENEURO.0026-19.2019.video.7

Movie 8. Representative time-lapse recording of mitochondrial dynamics in a growth cone of a *snph db* mutant embryo. Time-lapse video showing mitochondria (green, white) in a distal retinal axon and the growth cone (red) elongating along the optic tract of a *snph db* embryo. Images were acquired at 1 min intervals for 120 min. Note that the growth cone is pausing during the first minutes (00:00 to 00:47), after which it advances. Each frame is a confocal image stack maximal projection, lateral view, and anterior is on the left. Time stamp format: hours:minutes (hr:min). Scale bar, 5 μm. 10.1523/ENEURO.0026-19.2019.video.8

**Figure 8.**
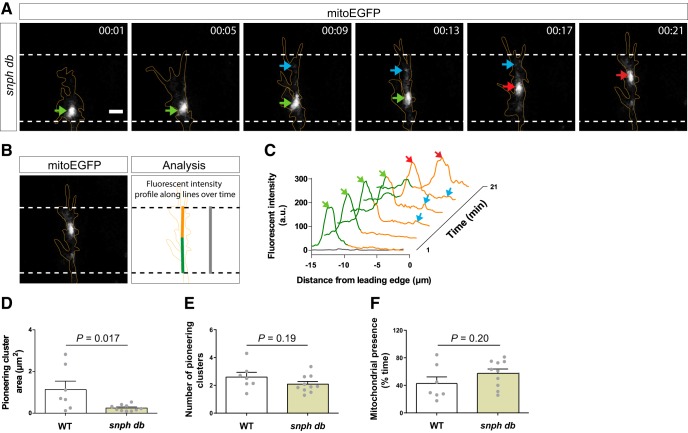
Syntaphilin contributes to the localization of pioneering mitochondrial clusters near the leading edge in advancing growth cones. ***A***, Representative time-lapse images of mitochondria (mitoEGFP, white) in an advancing growth cone (delineated in orange) in a *db* embryo. Like in WT (Fig. 3), the main mitochondrial cluster lags behind during growth cone advance while some pioneering mitochondria appear in close proximity to the leading edge. Lateral view, Confocal maximal projections. Scale bar, 3 μm. ***B***, ***C***, Fluorescent intensity profiles show the distribution of mitoEGFP fluorescence at various time points along the advancing growth cone shown in ***A***. Orange and green lines correspond to fluorescent intensities of mitochondria in the growth cone and the proximal growth cone, respectively. Arrows show peaks of fluorescence corresponding to localizations of mitochondria in the growth cone (see ***A***). ***D–F***, Quantification of pioneering mitochondrial cluster total area (***D***), number (***E***), and dynamics (percentage of time present during advance; ***F***). Pioneering mitochondrial clusters occupy a reduced area in *snph db* embryos compared with WT. Data from 12 independent experiments per genotype are shown as the mean ± SEM. Statistical analysis: unpaired *t* test.

Finally, in addition to the decreased mitochondrial occupancy of the growth cone in *db*, we observed some changes in pioneering mitochondrial clusters. As in WT, we detected pioneering clusters in every *db* growth cone that advanced (Fig. 8*A–C*). The number of clusters (Fig. 8*E*) and the amount of time they were present at the growth cone (Fig. 8*F*) did not vary; however, the total area occupied by pioneering mitochondrial clusters was significantly decreased in *db* compared with WT (Fig. 8*D*). Altogether, these observations demonstrate for the first time that Snphs contribute to mitochondrial clustering at the growth cone *in vivo*. Snphs play a role in anchoring mitochondria in the growth cone central domain, including both the main cluster and the pioneering mitochondria that dynamically appear along the leading edge of advancing growth cones.

### Mitochondrial docking at the growth cone by Syntaphilins is not required for axon outgrowth

As Snphs contribute to mitochondrial docking at the growth cone (Figs. 7*A–G*, 8), we then tested whether the loss of Snphs would affect axon elongation. Analysis of the total and central growth cone areas (Fig. 7*H*) and the number of filopodia (Fig. 7*I*) did not reveal any differences in growth cone morphology between WT and *snph db*. Moreover, retinal axon growth rate was similar between WT and *db* mutants (Fig. 7*J*), indicating that Snph-mediated mitochondrial docking at the growth cone is dispensable for axon elongation. Thus, we identified Snph as a mediator of mitochondrial docking at the growth cone in elongating axons *in vivo*, but this function plays only a minor role, if any, in axon growth.

## Discussion

By performing confocal live imaging in the zebrafish embryo, we provide the first detailed characterization of mitochondrial dynamics during growth cone behavior in elongating axons *in vivo*. We show that mitochondrial distribution at and trafficking toward the growth cone are coordinated with axon outgrowth, which is in agreement with previous *in vitro* observations (Morris and Hollenbeck, 1993; Sainath et al., 2017) and highlights that mitochondria might play a role in axon outgrowth and pathfinding. We further provide evidence that Snph contributes to mitochondrial docking at the growth cone. However, growth cone morphology and axon elongation are unaffected in *snph* db mutants, indicating that the direct anchoring of mitochondria to growth cone microtubules by Snph plays only a minor role in axon elongation.

An intriguing finding of our study is the localization of mitochondria along growth cone filopodia that is independent of Snph. Since filopodia dynamics are highly dependent on actin filament polymerization and turnover, this observation suggests that mitochondria might associate with the growth cone actin cytoskeleton. Mitochondria have indeed been shown to be able to attach to actin via the myosin 19 molecular motor (Shneyer et al., 2016). Interaction with myosin 19 positioned mitochondria into actin-rich filopodia of U-2 OS osteosarcoma cells following stress (Shneyer et al., 2017). A localization of mitochondria to leading edge lamellipodia, filopodia, and invadopodia of cancer cells has further been shown to play an important role in cancer cell migration and metastatic potential (Cunniff et al., 2016; Smith and Gallo, 2018). Whether the interaction among mitochondria, myosin 19, and actin is relevant in the growth cone remains unknown. Interestingly, mitochondria appeared along growth cone filopodia in a transient manner, suggesting that they might also be transported along the dynamic microtubules that explore the growth cone periphery and play an essential role in growth cone adhesion and turning (Buck and Zheng, 2002; Suter et al., 2004). Our observation of pioneering mitochondrial clusters at the leading edge of the growth cone during elongation further supports the hypothesis that mitochondria might frequently attach to the plus-end of microtubules. An intriguing consequence of mitochondrial targeting to microtubule plus ends in filopodia would be the possibility to predict the net direction of axon growth based on mitochondrial appearance. Unfortunately, our imaging conditions did not have the resolution required for correlating mitochondrial peripheral distribution with filopodia protrusion, retraction, or stabilization. It will be important in the future to optimize the imaging approaches to quantify fine aspects of mitochondrial behavior *in vivo* and test the possible interaction between mitochondria and the cytoskeleton in the growth cone. Interestingly, the presence of pioneering mitochondrial clusters close to the leading edge is at least partially Snph dependent, suggesting that the targeting of mitochondria to peripheral filopodia and to the leading edge rely on different mechanisms. While mitochondria might be transported on actin filaments or dynamic microtubules in the periphery, they may accumulate on the stable microtubules that “end” in the central area of the growth cone and be captured there by Snph and other factors.

The interdependence between mitochondrial transport and axon outgrowth that we observed is consistent with *in vitro* work demonstrating a net anterograde mitochondrial trafficking in growing axons that is abolished when axons encounter a physical barrier (Morris and Hollenbeck, 1993). We now establish such a correlation for the first time *in vivo* by comparing various parameters of mitochondrial transport in pausing versus advancing retinal axons. Together with the observation of a growth status-dependent mitochondrial distribution at the growth cone, our data indicate that mitochondrial dynamics in the distal axon and growth cone are coordinated with growth cone behavior and axon outgrowth *in vivo*. Vaarmann et al. (2016) previously showed that axon length could be increased by overexpressing PGC-1α, a central inducer of mitochondrial biogenesis, supporting the notion that mitochondria provide energy for axon growth. In line with this, pharmacological and genetic disturbance of the mitochondrial fission–fusion balance in cultured retinal neurons affected neurite length and caused neurite guidance errors (Steketee et al., 2012). On the other hand, other studies hinted at the ability of axons to grow with dysfunctional mitochondria (Yoon et al., 2012; Campbell et al., 2014). While depletion of the intermediate filament protein Lamin B2 caused defects in mitochondrial membrane potential, morphology, and transport, it did not affect retinal axon initial growth and guidance along the optic tract *in vivo* (Yoon et al., 2012). In a similar manner, lack of Kif5Aa, which transports mitochondria anterogradely into axons, causes a lack of mitochondria in distal peripheral axons that leads to degeneration. Yet, axons grow normally in *kif5Aa* zebrafish mutant embryos (Campbell et al., 2014). As it remains unclear which cellular processes are specifically dependent on mitochondrial respiration (Smith and Gallo, 2018), it might be conceivable that ATP production through glycolysis can, at least partially, sustain axon growth. Previous studies have even suggested that embryonic neurons predominantly rely on glycolytic ATP (Surin et al., 2012). As growth cone turning is regulated by local calcium levels (Gasperini et al., 2017), the ability of growth cone mitochondria to buffer calcium might be more important than its capacity to generate energy. Future work analyzing mitochondrial calcium dynamics at the growth cone *in vivo* might thus be highly interesting.

We identified Snph as a mediator of mitochondrial docking at the growth cone in elongating axons *in vivo*. This function is consistent with its previously established role in axonal mitochondrial stalling (Kang et al., 2008; Chen and Sheng, 2013). Yet, our data suggest that Snph does not play an important role in stalling mitochondria along the axon at this early developmental stage. The lack of difference in the number of mitochondria arriving at the growth cone in WT versus *snph db* axons suggests that Snph does not inhibit mitochondrial anterograde transport toward the growth cone as it does in the context of axon regeneration (Zhou et al., 2016). An increased halting of mitochondria along the axon over time is instead consistent with an increased expression of Snph in the brain during development, both in zebrafish in our study and in the mouse (Das et al., 2003; Kang et al., 2008). Alternatively, Snph localization and functions might be differently regulated in the axon shaft versus growth cone during development.

While the loss of Snphs reduced the number of mitochondria at the growth cone, it did not cause a complete depletion of mitochondria and did not affect axon growth. The remaining mitochondria at the growth cone might thus compensate for a lower density by increasing their activity. In this regard, it is noteworthy that *Snph* KO mice sciatic nerves show enhanced regrowth capacity after injury (Zhou et al., 2016). Cultured cortical neurons from these *Snph* KO mice displayed increased ATP content on axotomy (Zhou et al., 2016), but this difference in energy production was not observed in baseline conditions. Alternatively, the number of mitochondria remaining at the growth cone in the absence of Snph might be enough for normal axon development, especially considering that glycolytic ATP might have an important role in sustaining axon growth (Surin et al., 2012). These remaining mitochondria are likely stabilized at the growth cone central domain by other anchoring factors, of which the outer mitochondrial membrane GTPases Miro1 and 2 are especially good candidates. Miro proteins are indeed involved in mitochondrial trafficking along microtubules (Devine et al., 2016) and can arrest mitochondrial movement in a calcium-dependent manner (Saotome et al., 2008), which is particularly relevant at the growth cone where calcium signaling regulates pausing, extension, and turning (Sutherland et al., 2014; Gasperini et al., 2017). In that context, an elegant study recently demonstrated that growth inhibiting substrates such as MAG or chondroitin sulfate proteoglycans decrease mitochondrial axonal transport by promoting the deacetylation of Miro1 by HDAC6 in a calcium-dependent manner (Kalinski et al., 2019). Finally, Miros have also been discovered to play a role in mitochondrial trafficking and positioning along actin filaments (López-Doménech et al., 2018), which might regulate mitochondrial positioning along filopodia in the growth cone peripheral area. Future studies examining the role of Miros in mitochondrial docking at the growth cone *in vivo* will thus be of great interest to decipher how mitochondrial positioning is regulated during axonal development.

## Synthesis

Reviewing Editor: Mike Fainzilber, Weizmann Institute of Science Decisions are customarily a result of the Reviewing Editor and the peer reviewers coming together and discussing their recommendations until a consensus is reached. When revisions are invited, a fact-based synthesis statement explaining their decision and outlining what is needed to prepare a revision will be listed below. The following reviewer(s) agreed to reveal their identity: Eran Perlson.

In this study the authors manipulate expression of snph a and b in developing zebrafish embryos. They conclude that (a) syntaphilin contributes to mitochondrial immobilization at the growth cone; but also, that (b) syntaphilin is not required for proper growth cone morphology and axon elongation. In my previous comments I stated that the significance of the second finding hinges completely on the conclusiveness of the first. I currently feel that the additional data included in this version of the manuscript goes a long way to bolster both of the authors' conclusions. In this latest version, the authors have augmented the spatial analysis of mitochondrial occupancy inside of growth cones with volumetric measurements; they demonstrate that snph is indeed present in zebrafish growth cones and that Snph depletion does in fact result in more mitochondria exiting the growth cone. Taken together, the data presented successfully supports the hypothesis that syntaphilin does in fact contribute to mitochondrial immobilization at the growth cone. They also demonstrate that loss of Snph does not affect retinal axon development despite its importance for mitochondrial immobilization at growth cones. Taken together, these findings are interesting and worthy of publication in eNeuro.

There are a few minor issues that I feel the authors would do well to address:

1. Are the movies presenting the full resolution of the data? They seem a bit low-res compared to the images in the figures. Can they be improved?

2. In Figure 4C, the difference between the mitochondrial motility profile seen in Pausing vs. Advancing growth cones is marked as ** significance (i.e. p<0.01). However, in the statistics table the corresponding Two-way ANOVA is listed as producing P=0.8979. Is this a typo? Is the table referring to a different test than the one marked ** in the figure?

3. Most of the bar graphs in figure 7 (and in other figures) include all the data points, but panels E-H do not - is there a reason the authors chose to present datapoints only in a subset of panels?

4. In one bar graph in figure 7F (% retrograde), the authors chose to present the p-value itself (P=0.053) rather than the “n.s.” demarcation they use throughout the manuscript. I presume this is because the p statistic here just barely failed the customary but arbitrary 0.05 threshold for significance, but the authors still believe that the trend is biologically significant. In general, I feel it would benefit the manuscript to include the p-values for all 1 star and n.s. p-values in the actual figures rather than only in a separate table. This would be especially helpful in figure 7 since all other n.s. p-values fall very much farther from the 0.05 threshold; seeing the actual p-values would prevent the appearance of potential bias and make clear that the distinction between * and n.s is not merely 0.049 vs 0.051.
